# Supplementary material for: Spin-orbit coupling-enhanced valley ordering of malleable bands in twisted bilayer graphene on WSe2
Source: Nat Commun. 2023 Jul 8;14:4055. doi: 10.1038/s41467-023-39855-x (PMC10329678; doi:10.1038/s41467-023-39855-x)
Supplement: Supplementary file 1 — Supplementary Information [file 41467_2023_39855_MOESM1_ESM.pdf]

Spin-orbit coupling-enhanced valley ordering of malleable bands in twisted bilayer graphene on WSe_2

Saisab Bhowmik,[1] Bhaskar Ghawri,[2] Youngju Park,[3] Dongkyu Lee,[3,4] Suvronil Datta,[1] Radhika Soni,[1] K. Watanabe,[5] T. Taniguchi,[6] Arindam Ghosh,[2,7] Jeil Jung,[3,4] and U. Chandni[1]

[1]*Department of Instrumentation and Applied Physics, Indian Institute of Science, Bangalore, 560012, India*
[2]*Department of Physics, Indian Institute of Science, Bangalore, 560012, India*
[3]*Department of Physics, University of Seoul, Seoul 02504, Korea*
[4]*Department of Smart Cities, University of Seoul, Seoul 02504, Korea*
[5]*Research Center for Functional Materials, National Institute for Materials Science, Namiki 1-1, Tsukuba, Ibaraki 305-0044, Japan*
[6]*International Center for Materials Nanoarchitectonics, National Institute for Materials Science, Namiki 1-1, Tsukuba, Ibaraki 305-0044, Japan*
[7]*Centre for Nano Science and Engineering, Indian Institute of Science, Bangalore 560 012, India*

SUPPLEMENTARY INFORMATION

I. EXPERIMENTAL RESULTS

Supplementary Note 1. Additional data for the pair of contacts reported in the main text

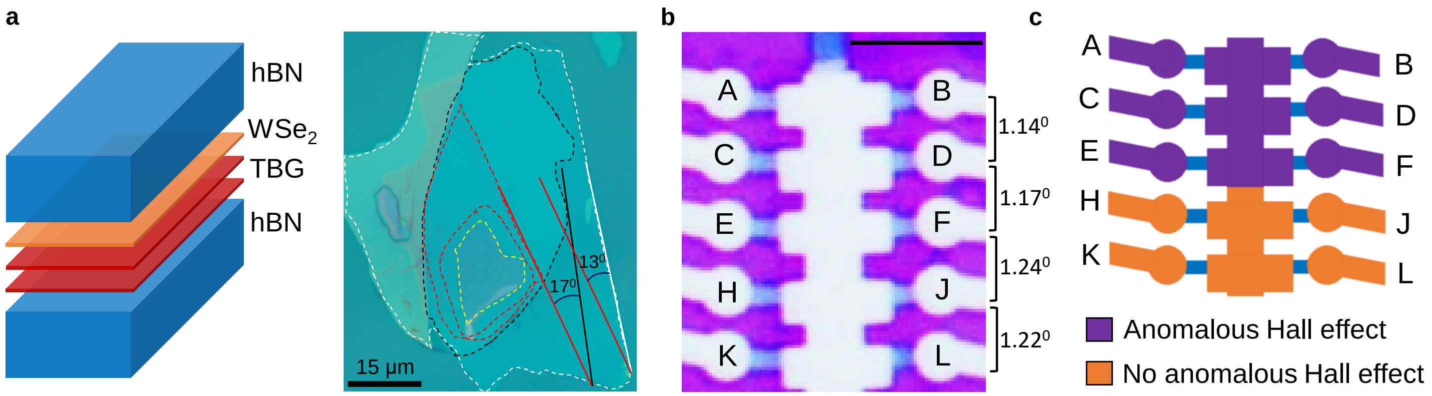

Supplementary Fig. 1. TBG-WSe_2 device. **a.** The schematic of the device shows a multilayer WSe_2 between top hBN and TBG (left). The optical image of the heterostructure is shown in right, with constituent layers denoted by different colors: top and bottom graphene (red), WSe_2 (yellow), top hBN (black), and bottom hBN (white). The graphene layers in TBG are misaligned with both top and bottom hBN (shown by straight lines along the edges of graphene and hBN). **b.** Optical micrograph of the device with the edge contacts labelled from A to L. The twist angles measured between the adjacent contacts are indicated. Throughout the main text, we show data for R_{xx} measured between contacts D and F, and R_{xy} measured between contacts C and D. The scale bar denotes 5 μm.**c.** Anomalous Hall effect is observed for three pairs of contacts (A-F) denoted by purple colour, while the other four contacts (H, J, K, and L) denoted by orange colour do not exhibit any hysteresis.

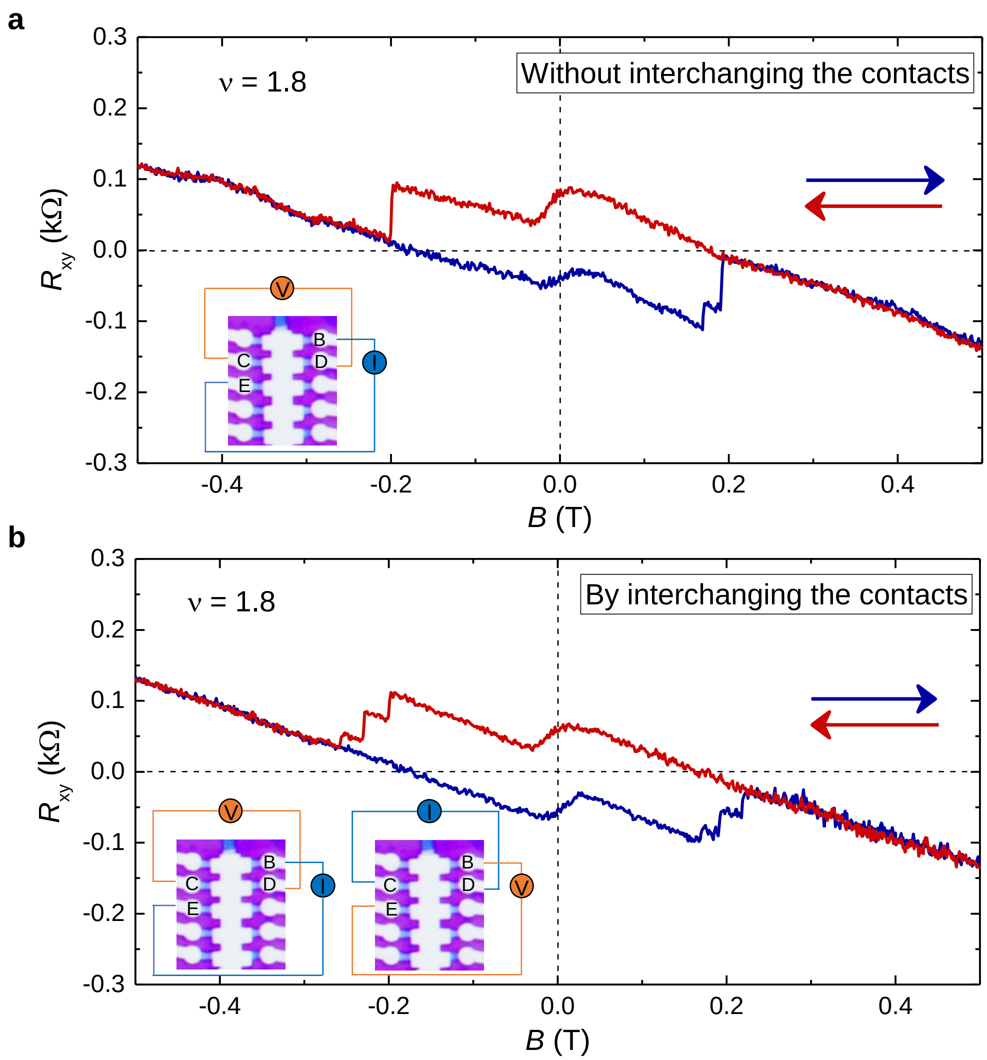

Supplementary Fig. 2. Hall resistance R_{xy} measured as a function of magnetic field B using Onsager reciprocity theorem [1, 2]. a. Measurement of R_{xy} for B-field sweep at $\nu = 1.8$ by driving current from contact E to B and measuring voltage between contacts C and D. Blue and orange colours are used for current and voltage probes (inset). Blue and red colours in the data denote the direction of B-field sweep. **b.** First, R_{xy} is measured as a function of B-field sweep by passing current from contacts E to B and measuring voltage between contacts C and D (bottom left inset). Second, R_{xy} is measured as a function of B-field sweep by passing current from contacts C to D and measuring voltage between contacts E and B (bottom right inset). The antisymmetrized R_{xy} is calculated using $R_{xy} = (R_{EBCD} - R_{CDEB})/2$. The width and shape of the hysteresis obtained by measuring R_{xy} using the above discussed two methods do not match perfectly. The transverse voltage in an out-of-plane B relies on the fact that R_{xy} is antisymmetric in B and it is accurately determined using $R_{xy} = \{R_{xy}(B) - R_{xy}(-B)\}/2$. However, this method does not perfectly hold true for a ferromagnet where the dynamics of magnetic domains throughout the device may have different responses to the magnetic field. For the precise determination of R_{xy} below coercive field we have used Onsager reciprocity theorem and the ferromagnetism data presented in the paper are obtained using the reciprocity theorem, as detailed in (b).

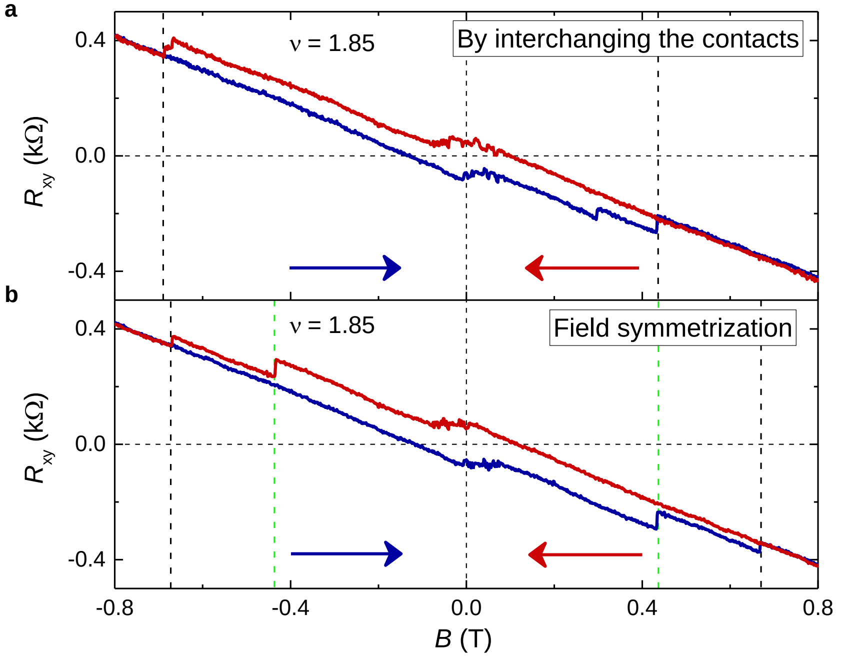

Supplementary Fig. 3. Comparison of R_{xy} using Onsager antisymmetrization and field antisymmetrization. **a.** R_{xy} as a function of B measured by interchanging current and voltage probes as discussed in Fig. S2. **b.** To compare R_{xy} data resulting from different measurement configurations, we show the hysteresis at $\nu = 1.85$ obtained from field antisymmetrized $R_{xy}(\overleftarrow{B}) = \{R_{xy}(\overleftarrow{B}) - R_{xy}(\overrightarrow{-B})\}/2$, $R_{xy}(\overrightarrow{B}) = \{R_{xy}(\overrightarrow{B}) - R_{xy}(\overleftarrow{-B})\}/2$ where $\overleftarrow{B}$ and $\overrightarrow{B}$ denote two opposite direction of B sweep. In this method, a significant reduction in the width of the hysteresis in R_{xy} for $0.44 \lesssim |B| \lesssim 0.67$ (shown by dashed green lines) indicates the asymmetry between positive and negative coercive fields. However, the method discussed in **a.** better captures the dynamics of magnetic domains, which are asymmetric in magnetic field. Therefore, we have used the Onsager anti-symmetrization method for the characterization of hysteresis in our device.

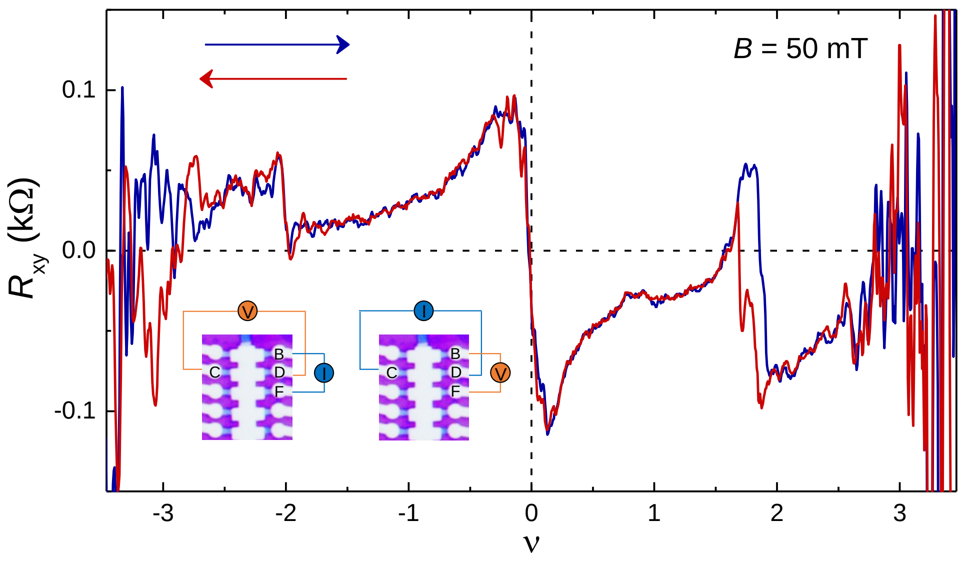

Supplementary Fig. 4. Low-field Hall data for two opposite sweep directions of carrier density at $T = 0.3$ K. R_{xy} plotted as a function of ν at $B = 50$ mT shows a hysteresis loop with respect to density only in the vicinity of $\nu = 2$. Here, R_{xy} is calculated using Onsager reciprocity theorem as discussed in Fig. S2.

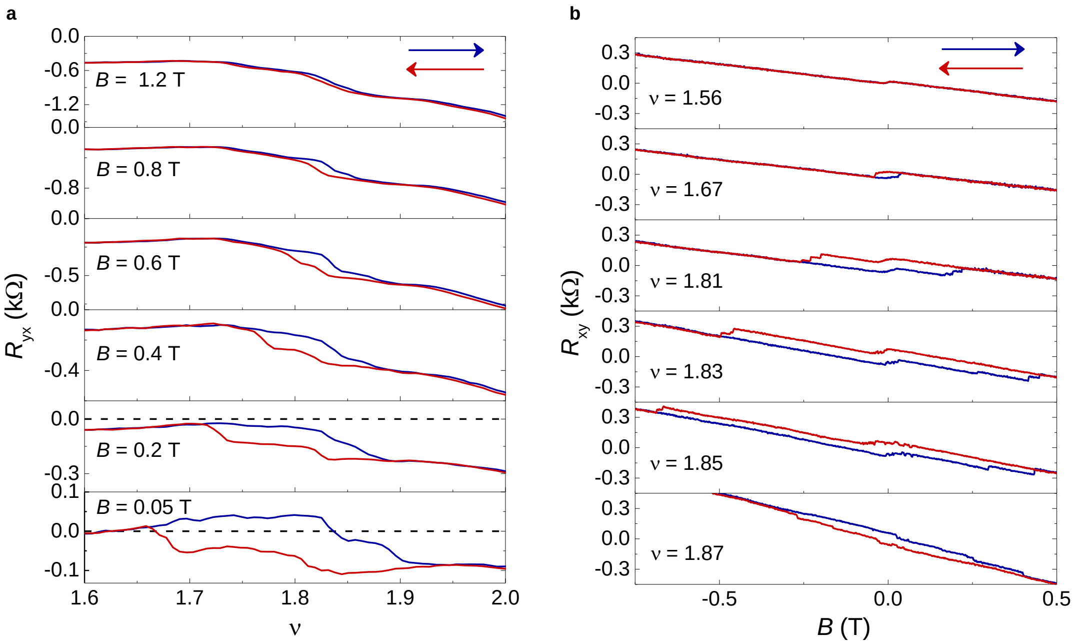

Supplementary Fig. 5. The evolution of hysteresis in density and magnetic field. **a.** R_{yx} shows hysteretic behaviour when the density is swept back and forth at low magnetic fields up to 1.2 T. The hysteresis is strongly suppressed with increasing B. R_{yx} shows zero crossings only at $B = 0.05$ T and remains positive at other fields. **b.** The hysteresis in R_{xy} with repect to out of plane B-field in plotted for different ν below $\nu = 2$. The width of the hysteresis in B is highly tunable with density and the reversal of the hysteresis in clearly seen.

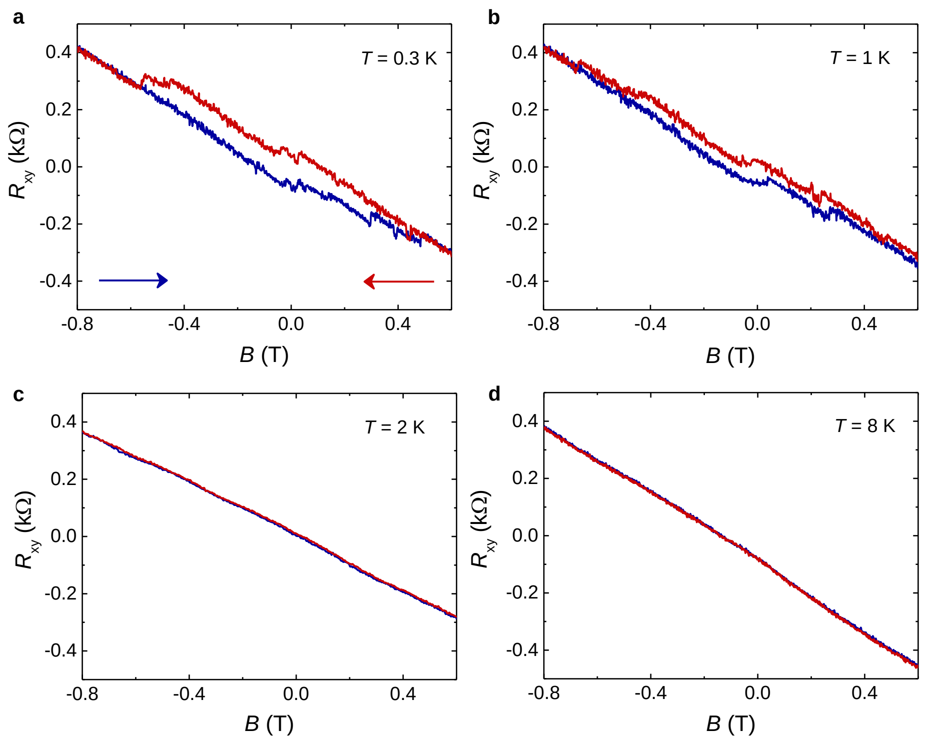

Supplementary Fig. 6. Temperature dependence of the hysteresis a.-d. R_{xy} as a function of B for two opposite sweep directions is plotted for different temperatures. The hysteresis becomes weaker with increasing temperature and disappears at $T = 2$ K.

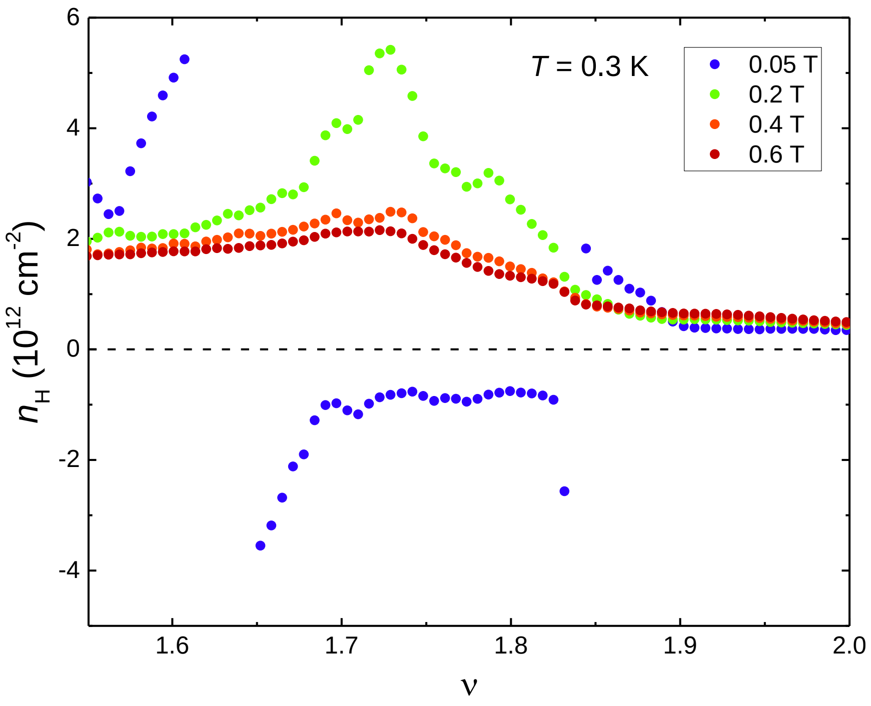

Supplementary Fig. 7. Fermi surface reconstructions at $T = 0.3$ K. Hall density n_H plotted as a function of ν for $B = 0.05, 0.2, 0.4$ and 0.6 T at $T = 0.3$ K. Lifshitz transitions and reset of charge carriers are observed for $B = 0.05T$, whereas only reset of charge carriers occur for $B = 0.2 - 0.6$ T.

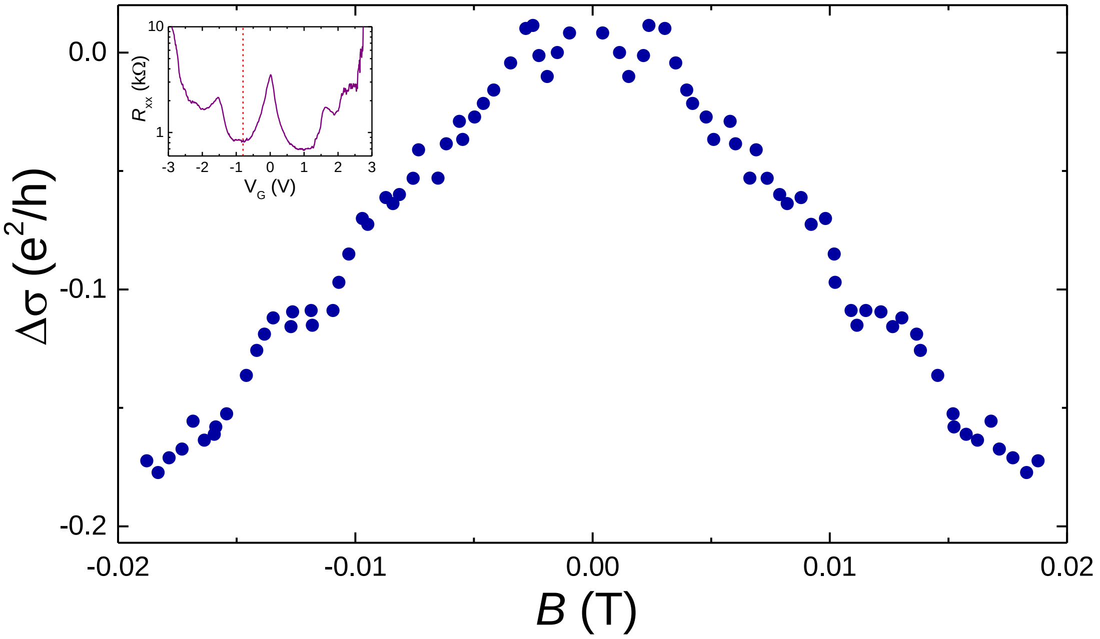

Supplementary Fig. 8. Weak antilocalization data at $T = 0.3$ K. The change in longitudinal magneto-conductivity $\Delta\sigma$ with respect to $B = 0$ mT data, is plotted as a function of B from -20 mT to 20 mT for a gate voltage $V_G = -0.8$ V (shown by the dashed red line in the inset). A peak in $\Delta\sigma$ at $B = 0$ mT indicates weak antilocalization and hence, a finite spin-orbit coupling in graphene layers proximity coupled to WSe_2. The estimation of spin-orbit coupling strength requires the fitting of the theoretical formula for $\Delta\sigma$ that depends on four scattering time scales in the weak antilocalization regime [3]. Although this model works for graphene/transition metal dichalcogenide systems [4], a more detailed calculation is needed to obtain an exact model for TBG, for a quantitative comparison. At this stage, we can confirm the presence of spin-orbit coupling qualitatively from the peak in $\Delta\sigma$ at $B = 0$.

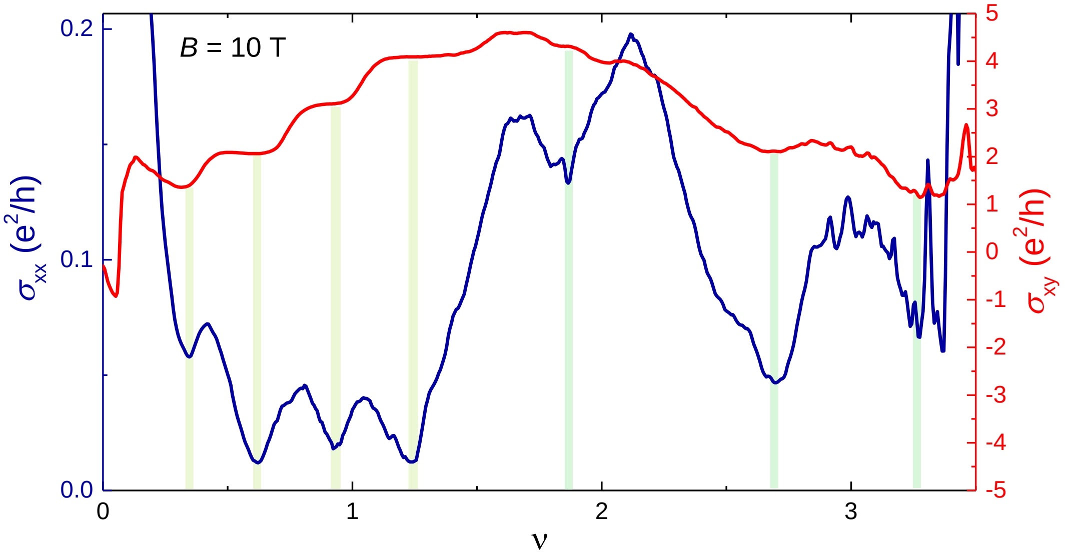

Supplementary Fig. 9. Hall conductivity σ_{xy} and longitudinal conductivity σ_{xx} at $B = 10$ T. σ_{xy} shows plateaus as $\sigma_{xy} = Ce^2/h$ associated with the minima in σ_{xx}. Different color bars (yellow for the CNP, green for $\nu = 1, 2, 3$) have been used to show the quantized states emanating from several partial fillings of flat bands.

Supplementary Note 2. Ferromagnetism in additional sets of contacts

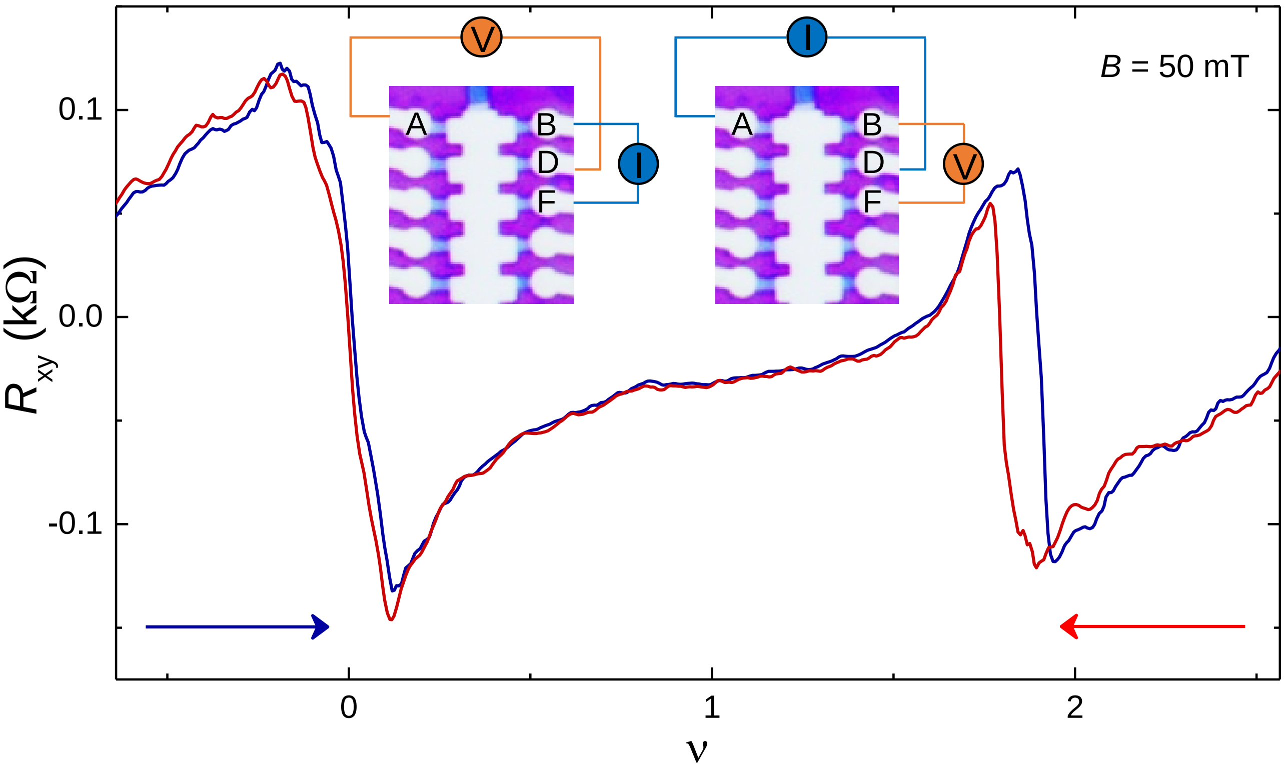

Supplementary Fig. 10. Low-field Hall data for two opposite sweep directions of carrier density. R_{xy} plotted as a function of ν at $B = 50$ mT shows a hysteresis loop with respect to density only in the vicinity of $\nu = 2$ that is very similar to the data in Fig. S4. Here, R_{xy} is calculated using Onsager reciprocity theorem with the current and voltage probes shown in the inset. Note that these measurements using additional contacts were performed at $T = 0.3$ K in a different thermal cycle compared to the measurements shown in section A and the main text.

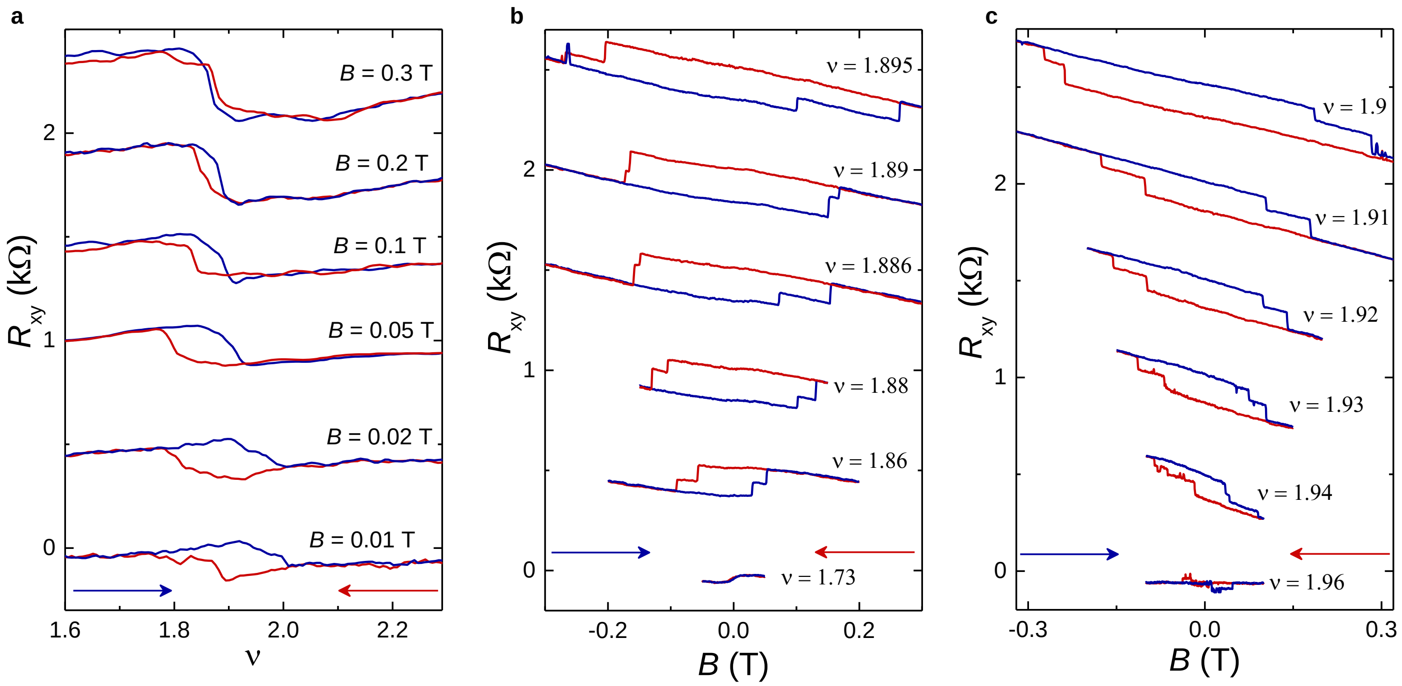

Supplementary Fig. 11. Hysteresis with respect to density and magnetic field. **a.** Hysteresis in R_{xy} with respect to density sweep. R_{xy} is measured using the set of contacts as mentioned in Fig. S10. The hysteresis is strongly suppressed with increasing B and disappears at $B = 0.3$ T. The hysteresis loops are shifted vertically by 0.5 kΩ for better clarity. **b.-c.** The hysteresis in R_{xy} with respect to out of plane B-field is plotted for different ν below $\nu = 2$. The width of the hysteresis in B becomes larger as the density is increased from $\nu = 1.73$. The reversal of the hysteresis takes place at $\nu = 1.9$, further to which the hysteresis loop reduces and disappears at $\nu = 1.96$.

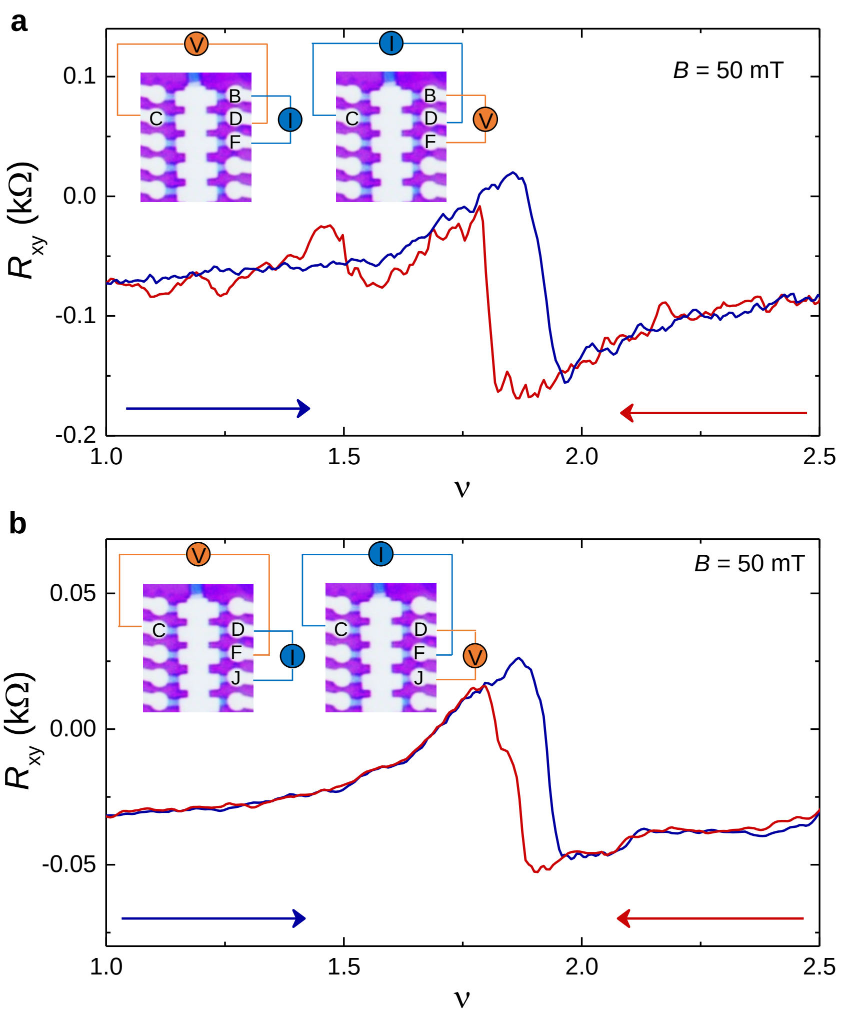

Supplementary Fig. 12. Low-field hysteresis for additional contacts. **a.** R_{xy} measured in a later thermal cycle using the original contact configuration shown in the main text. We do not find any significant change due to thermal cycling. **b.** A similar hysteresis loop is observed for a different set of contacts shown in the inset.

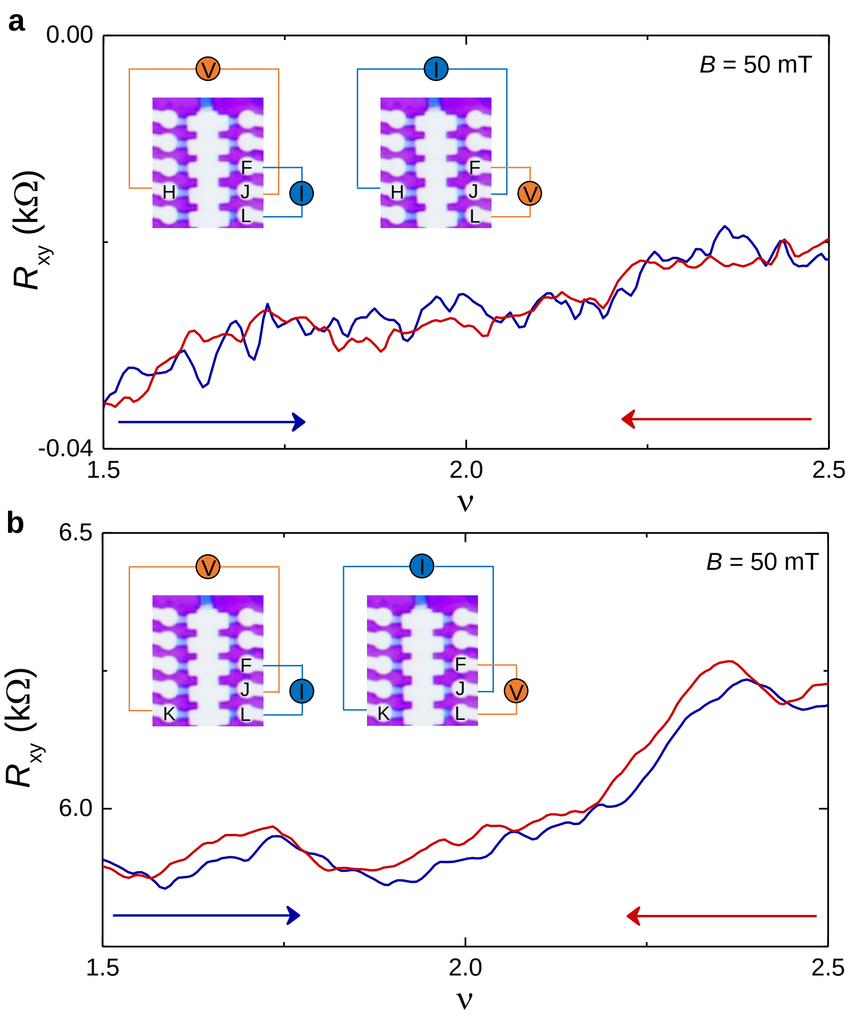

Supplementary Fig. 13 Absence of anomalous Hall effect in the lower sets of contacts. **a.-b.** No evidence for hysteresis is found using the four lower sets of contacts shown in the insets. We speculate that the higher twist angle in this region of the device leads to suppression of the anomalous Hall effect.

Supplementary Note 3. AHE and Chern insulator at zero and high B-field limits, respectively

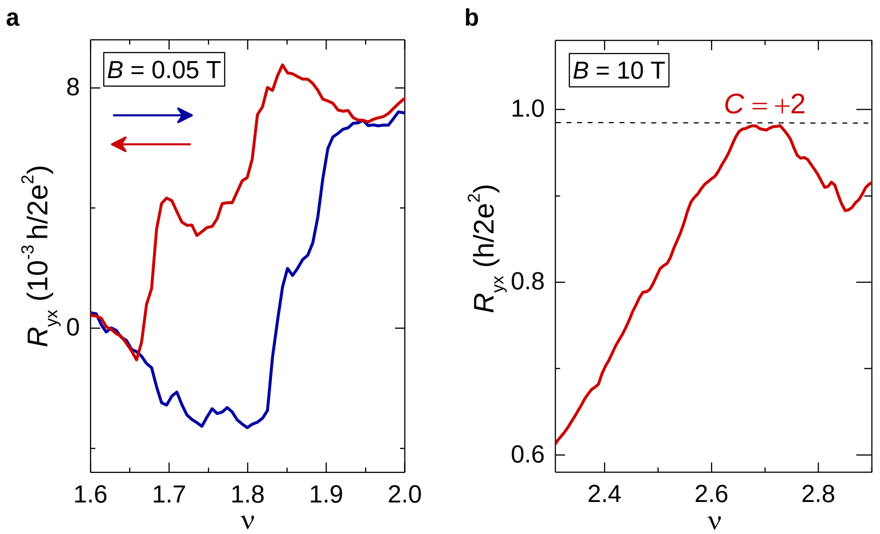

Supplementary Fig. 14. Comparison of transport data between zero and high B limits. **a.** R_{xy} shows a hysteresis at $B = 0.05$ T when the direction of density sweep is reversed (red and blue arrows indicate two opposite directions). The magnitude of R_{xy} is much lower than the quantized value ($h/2e^2$).**b.** R_{xy} plotted as a function of ν at $B = 10$ T shows a quantized plateau originating from $\nu = 2$.

A. Supplementary Note 4. Possible ground states at $\nu = 2$

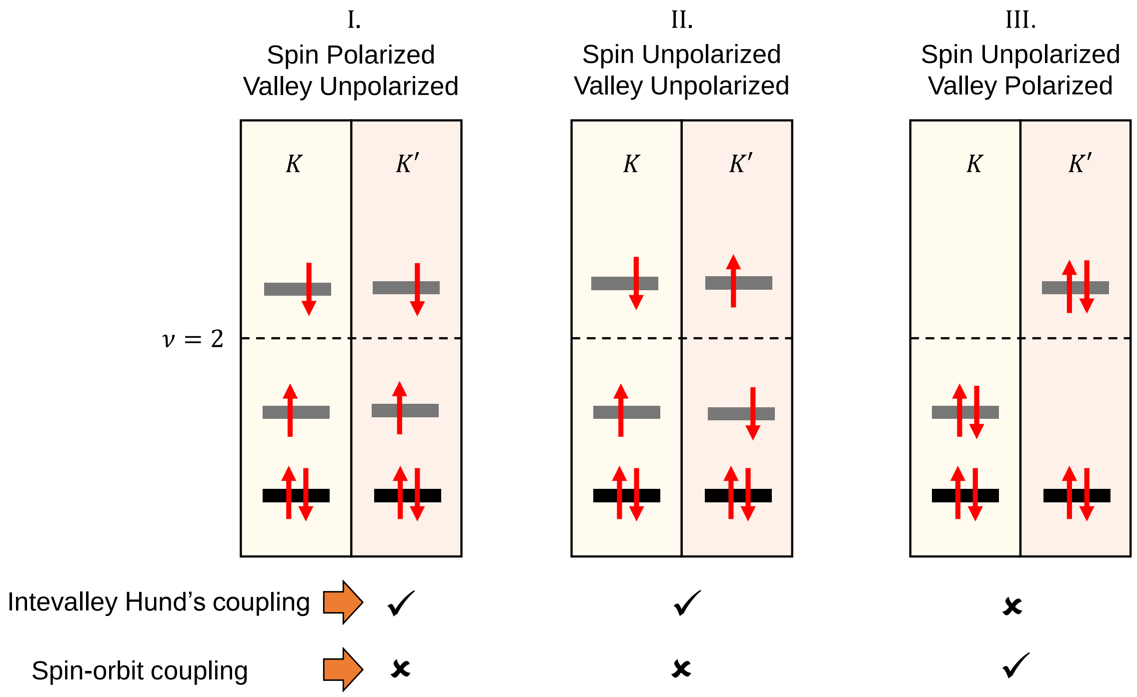

Supplementary Fig. 15. **Ground states at** $\nu = 2$. In TBG without WSe_2, at $\nu = 2$ three possible ground states are degenerate in energy. A small inter-valley Hund's coupling lifts the degeneracy and favours either of the first two states (spin/valley unpolarized, and spin-polarized, valley unpolarized). Hence, ferromagnetism is not expected at $\nu = 2$ since the ground state is valley unpolarized. However, proximity-induced spin-orbit coupling in TBG by WSe_2 leads to a valley imbalance, and the third state is favoured. As a result, the system exhibits ferromagnetism at $\nu = 2$.

II. SPIN-POLARIZED PHASE IN MEAN-FIELD HUBBARD MODEL CALCULATION

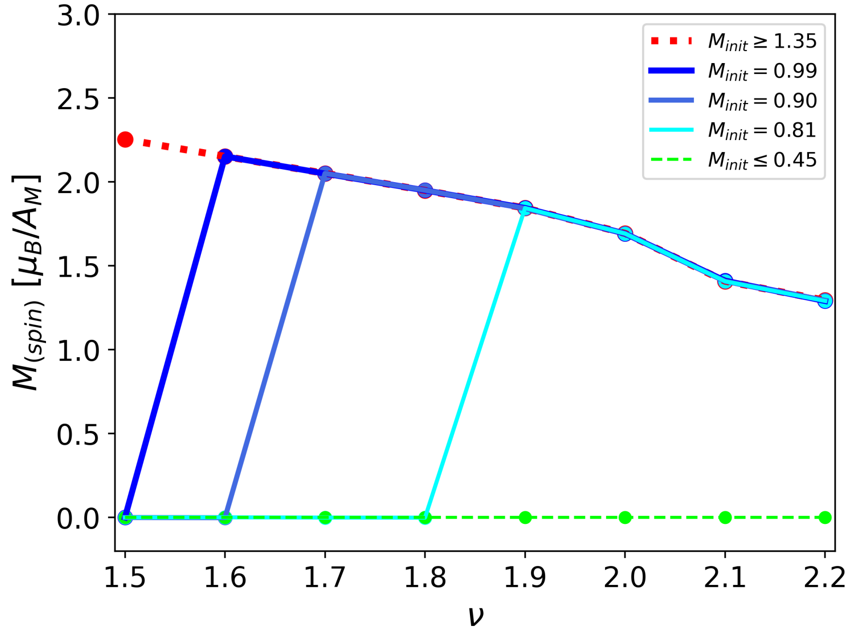

Supplementary Fig. 16. Spin magnetization of converged states of Hubbard model calculation. The total spin magnetic moments of the moiré unit-cell was obtained from self-consistent spin density. From $M_{init} \leq 0.45$ (Light green) and $M_{init} \geq 1.35$ (Red), the non-magnetic phase and the ferromagnetic phases are clearly identified. It is confirmed from the trend of magnetization change that the two phases become closer as the filling increases. In $M_{init} = 0.81, 0.9, 0.99$ case, the states are separated between $\nu = 1.6$ and $\nu = 1.9$ and converged to the two different phases manifested in lower and higher initial magnetization cases.

A mean-field Hubbard model of the system introduces the spin-polarizes phase, which further enhances the degeneracy lifting and, therefore, the valley polarization. The initial spin densities were assumed as uniformly distributed spin-ordered states of total magnetization in a unit moiré cell $M_{init} = 0 \sim 4.5[\mu_B/A_{Moire}]$. Interestingly, two stable convergence states of the non-magnetic and ferromagnetic phases were revealed according to the initial spin density. In addition, it was shown that the convergence phase transition at $\nu = 1.7$ under the initial magnetic condition of $M_{init} = 0.9$.

The tight-binding Hamiltonian of graphene on WSe_2 for the Hubbard model calculation inherits the proximity spin-orbit coupling effect and sublattice-dependent potentials due to the contacting layer. Following the formulations presented in Refs. [5] we consider the Hamiltonian

$$\begin{aligned} H =& H_0 + \sum_{i \in Bot, \sigma} \Delta \xi^i c^\dagger_{i,\sigma} c_{i,\sigma} \\ &+ \frac{2i}{3} \sum_{\langle i,j \rangle}^{Bot} \sum_{\sigma,\sigma'} \lambda_R c^\dagger_{i,\sigma} c_{j,\sigma'} (s^x_{\sigma,\sigma'} d^y_{i,j} - s^y_{\sigma,\sigma'} d^x_{i,j}) \\ &+ \frac{i}{3\sqrt{3}} \sum_{\langle\langle i,j \rangle\rangle}^{Bot} \sum_{\sigma,\sigma'} \lambda^i_I c^\dagger_{i,\sigma} c_{j,\sigma'} \nu_{i,j} s^z_{\sigma,\sigma'} \\ &+ \frac{2i}{3} \sum_{\langle\langle i,j \rangle\rangle}^{Bot} \sum_{\sigma,\sigma'} \lambda^i_{PIA} c^\dagger_{i,\sigma} c_{j,\sigma'} (s^x_{\sigma,\sigma'} d^y_{i,j} - s^y_{\sigma,\sigma'} d^x_{i,j}) \\ &+ \sum_{i,\sigma,\sigma' \neq \sigma} U \rho_{i,\sigma'} c^\dagger_{i,\sigma} c_{i,\sigma} \end{aligned} \tag{1}$$

Here, H_0 is non-interacting hopping model for magic-angle twisted bi-layer graphene with relaxation effects [6], and the second to fifth terms express spin-orbit interactions applied to the bottom layer graphene to effectively reflect the WSe_2 layers[5]. The 1.7° commensurate tBG structure is used for this calculation with rescaling parameter $S' = 1.608$ corresponding with the magic angle. Δ is the induced orbital staggered potential, λ_I is the intrinsic spin-orbit couplings, λ_R is the Rashba spin-orbit coupling and λ_{PIA} is the pseudospin-inversion-asymmetry(PIA) spin-orbit terms. We used $\Delta = 0.54$ meV and $\lambda_R = 0.56$ meV. In the case of λ_I and λ_{PIA}, the values are different for each sublattice and are $\lambda_I^A{=}{-1.22}$ meV, $\lambda_I^B{=}1.16$ meV,

$\lambda^A_{PIA} = -2.69$ meV, $\lambda^B_{PIA} = 2.54$ meV. The last term considers the mean-field Hubbard model. The spin density $\rho_{i,\sigma}$, considering the neutral density n^0 obtained from the Non-interacting Hamiltonian, is defined as $\rho_{i,\sigma} \equiv \langle c^\dagger_{i,\sigma} c_{i,\sigma} \rangle - n^0_{i,\sigma}$. The self-consistent calculations were performed on 5×5 Monkhorst-Pack grids for U = 5 eV.

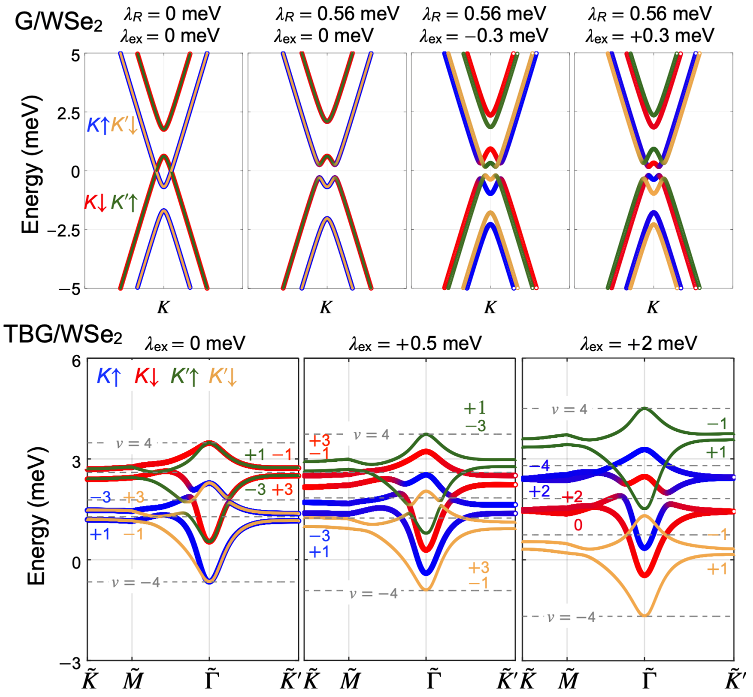

Supplementary Fig. 17. Exchange-field induced valley split bands of graphene (G) on WSe_2 and TBG on WSe_2 calculated within the continuum model, including SOC. For TBG, we assume the magic twist angle $\theta = 1.05°$ with a Fermi velocity of $\upsilon_F = 1 \times 10^6$ m/s. The SOC parameters λ^A_I, λ^B_I, and λ_R, λ^A_{PIA}, λ^B_{PIA} use the default values proposed in Ref. [5] except when they are defined explicitly. The proximity SOC induced by the WSe_2 layer lifts the degeneracy of the spin up and down bands within each valley, but the intervalley degeneracy between opposite spins of $K\uparrow$ ($K\downarrow$) and $K'\downarrow$ ($K'\uparrow$) bands is maintained. An exchange field $\lambda_{\rm ex}$ that reflects the magnitude and sense of the spin polarisation breaks the remaining degeneracy and can easily alter the ordering of the spin-valley flavours.

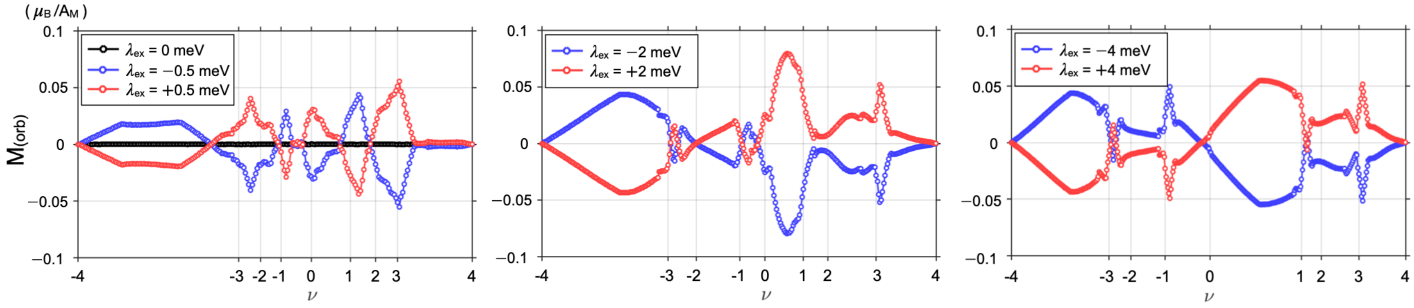

Supplementary Fig. 18. Orbital magnetization of the TBG-WSe_2 bands in Supplementary Fig. 17 using continuum model. In the absence of the exchange field ($\lambda_{\rm ex} = 0$), the orbital magnetization from the K and K' valleys cancel each other leading to the zero total orbital magnetization (black). For the finite $\lambda_{\rm ex} = \pm 0.5,\ 2,\ 4$ meV, the orbital magnetization of each valley remains finite where the sign flip of the slope along with the band filling v indicates the topological phase transition from positive to negative Chern number.

III. COMPARISON WITH PREVIOUS REPORTS ON AHE IN TBG SYSTEMS

Manuscript reference	Filling	Number of devices	Pair of contacts	Area of the ferromagnetic region (in μm^2)	Area of the ferromagnetic region/Total area of the device (in %)
10	3	1	2	9	100
9	3	1	2	18	34
22	1	1	2	17	25
20	2	2	2	13	47
21	2	1	3	28	58
21	-2	1	1	4	10
45	1	1	1	38	28
Our work	2	1	3	15	60

Supplementary Table 1. The table shows the number of devices measured in the six different works that have demonstrated AHE at different integer fillings in TBG. The number of contacts with the effective area of samples showing AHE are included based on the data presented in the literature. About 60% of the total area of our sample exhibits AHE. In Ref. [5] the total area of the device is the smallest ($\approx 9\ \mu\text{m}^2$) among all the reports in this table. Therefore 100% does not necessarily indicate strong and uniform bulk ferromagnetism.

SUPPLEMENTARY REFERENCES

[1] Sample, H., Bruno, W., Sample, S. & Sichel, E. Reverse-field reciprocity for conducting specimens in magnetic fields. *Journal of Applied Physics* **61**, 1079–1084 (1987).

[2] Serlin, M. *et al.* Intrinsic quantized anomalous Hall effect in a moiré heterostructure. *Science* **367**, 900–903 (2020).

[3] McCann, E. & Fal'ko, V. I. $z \to -z$ symmetry of spin-orbit coupling and weak localization in graphene. *Phys. Rev. Lett.* **108**, 166606 (2012).

[4] Wakamura, T. *et al.* Spin-orbit interaction induced in graphene by transition metal dichalcogenides. *Phys. Rev. B* **99**, 245402 (2019).

[5] Gmitra, M., Kochan, D., Högl, P. & Fabian, J. Trivial and inverted dirac bands and the emergence of quantum spin hall states in graphene on transition-metal dichalcogenides. *Phys. Rev. B* **93**, 155104 (2016).

[6] Leconte, N., Javvaji, S., An, J., Samudrala, A. & Jung, J. Relaxation effects in twisted bilayer graphene: A multiscale approach. *Phys. Rev. B* **106**, 115410 (2022).
